# PET/CT in therapy control of infective native aortic aneurysms

**DOI:** 10.1038/s41598-021-84658-z

**Published:** 2021-03-03

**Authors:** Lars Husmann, Martin W. Huellner, Nadia Eberhard, Bruno Ledergerber, Marisa B. Kaelin, Alexia Anagnostopoulos, Ken Kudura, Irene A. Burger, Carlos-A. Mestres, Zoran Rancic, Barbara Hasse

**Affiliations:** 1 Department of Nuclear Medicine, University Hospital Zurich/University of Zurich, Raemistrasse 100, 8091 Zurich, Switzerland; 2 Division of Infectious Diseases and Hospital Epidemiology, University Hospital Zurich/University of Zurich, Zurich, Switzerland; 3 Clinic for Cardiac Surgery, University Hospital Zurich/University of Zurich, Zurich, Switzerland; 4 Clinic for Vascular Surgery, University Hospital Zurich/University of Zurich, Zurich, Switzerland

**Keywords:** Aneurysm, Molecular medicine

## Abstract

Infective native aortic aneurysms (INAA) are aneurysms arising from infection of the aortic wall. Treatment is demanding with 5-year survival rates between 53 and 55%. The aim of our study was to evaluate the usefulness of ^18^F-fluorodeoxyglucose positron emission tomography/computed tomography (PET/CT) in the long-term monitoring of patients with proven INAA. Fifty-three PET/CT were performed in 15 patients with INAA in this single-center retrospective cohort study and retrospective analysis of prospectively collected Vascular Graft Cohort Study (VASGRA) data. Median metabolic activity (as measured by maximum standardized uptake value, SUVmax) of the aneurysms at the initial PET/CT was high (6.8 (IQR 5.7–21.8)), and lower at the last PET/CT prior to the end of antimicrobial therapy (3.9 (IQR 2.7–6.8); n = 11) as well as in the first PET/CT after the end of the treatment (3.9 (IQR 3.0–4.4);n = 6). Compared to the course of C-reactive protein alone, PET/CT provided different (> 20% difference in trend) or altering (opposed trend) information on the course of disease in at least 14 comparisons (56%) in 11 patients (73%). The one-year and five-year freedom from all-cause lethality was 92% (95% confidence interval 57%-99%). As compared to the course of C-reactive protein, PET/CT provides different and occasionally altering information in therapy control of INAA.

## Introduction

Surgical and medical treatment of infective native aortic aneurysms (INAA) is demanding and the imminent rupture of the arterial vessel wall requires immediate surgical care^1,2^. Open surgical treatment includes resection of the aneurysm, extensive local debridement, and revascularization by in situ reconstruction or extra-anatomic bypass^3,4^. In recent years, there is increasing evidence that endovascular aortic repair (EVAR) of INAA may be an equivalent treatment option^5,6^, especially in cases where the anatomic location of the aneurysm precludes open surgical repair. Endovascular treatment of INAA frequently leads to secondary vascular graft infections (VGI). Treatment of secondary graft infections generally involves long-term antimicrobial therapy^7,8^ depending on the respective microorganisms, graft location, and involved graft material. Based on the heterogeneity of these factors, there are still many uncertainties with regard to the type and length of antimicrobial therapy^7^.

^18^F-fluorodeoxyglucose positron emission tomography/computed tomography (PET/CT) can be used to assess the treatment response in VGI. Its impact on patient management has been investigated in three preliminary studies^9–11^, with promising results. To date, PET/CT has not been investigated in treatment response assessment of INAA. Thus, the aim of our study was to evaluate the usefulness of PET/CT in the long-term monitoring of patients with proven INAA.

## Methods

### Study design and definitions

Eligible participants included (a) prospectively acquired patients aged 18 years or older with proven INAA and open and/or endovascular surgery enrolled in the Vascular Graft Cohort Study (VASGRA), or (b) retrospectively acquired patients with proven INAA, who were examined at least twice with PET/CT between the years 2005 and 2018. The study was approved by the local ethics committee, namely the Kantonale Ethikkomission Zürich (protocol number 2018-01904), and we obtained written informed consent from all participants who were either prospectively enrolled or examined between the years 2016 and 2018; for subjects scanned between the years 2005 and 2015, written informed consent was waived due to retrospective inclusion by the local ethics committee, namely the Kantonale Ethikkomission Zürich (protocol number 2018-01904). All procedures were performed in accordance with the 1964 Helsinki declaration and its later amendments or comparable ethical standards.

Diagnosis of INAA was made in an overall appraisal of clinical presentation (pain, fever, sepsis), laboratory (positive microbiological culture of aortic/aneurysmatic wall, presence of bacteria in thrombus or blood culture; elevation of inflammatory markers such as C-reactive protein and leucocytes) and imaging^5^. Diagnosis of secondary VGI due to placement of stentgrafts relied on the MAGIC criteria^12^.

In all prospectively enrolled patients, criteria for the termination of antimicrobial therapy were a combination of the absence of clinical features of infection, normal CRP, and reduced metabolic activity in PET/CT. Cure of INAA was defined as a combination of absence of clinical features, normal CRP, and reduced metabolic activity in PETCT at least three months after termination of antibiotic therapy (the latter only in the prospectively enrolled patients).

For retrospective inclusion of patients, we performed a retrospective chart review in all patients with suspected INAA, who were examined with PET/CT between the years 2005 and 2018, if the term “mycotic aneurysm”, “infective aneurysm”, or “infected aneurysm” was mentioned in the written PET/CT report, and was found to refer to an aneurysm of the thoracic, abdominal or pelvic arteries.

### PET/CT examinations and patient follow-up

**Figure 1:**
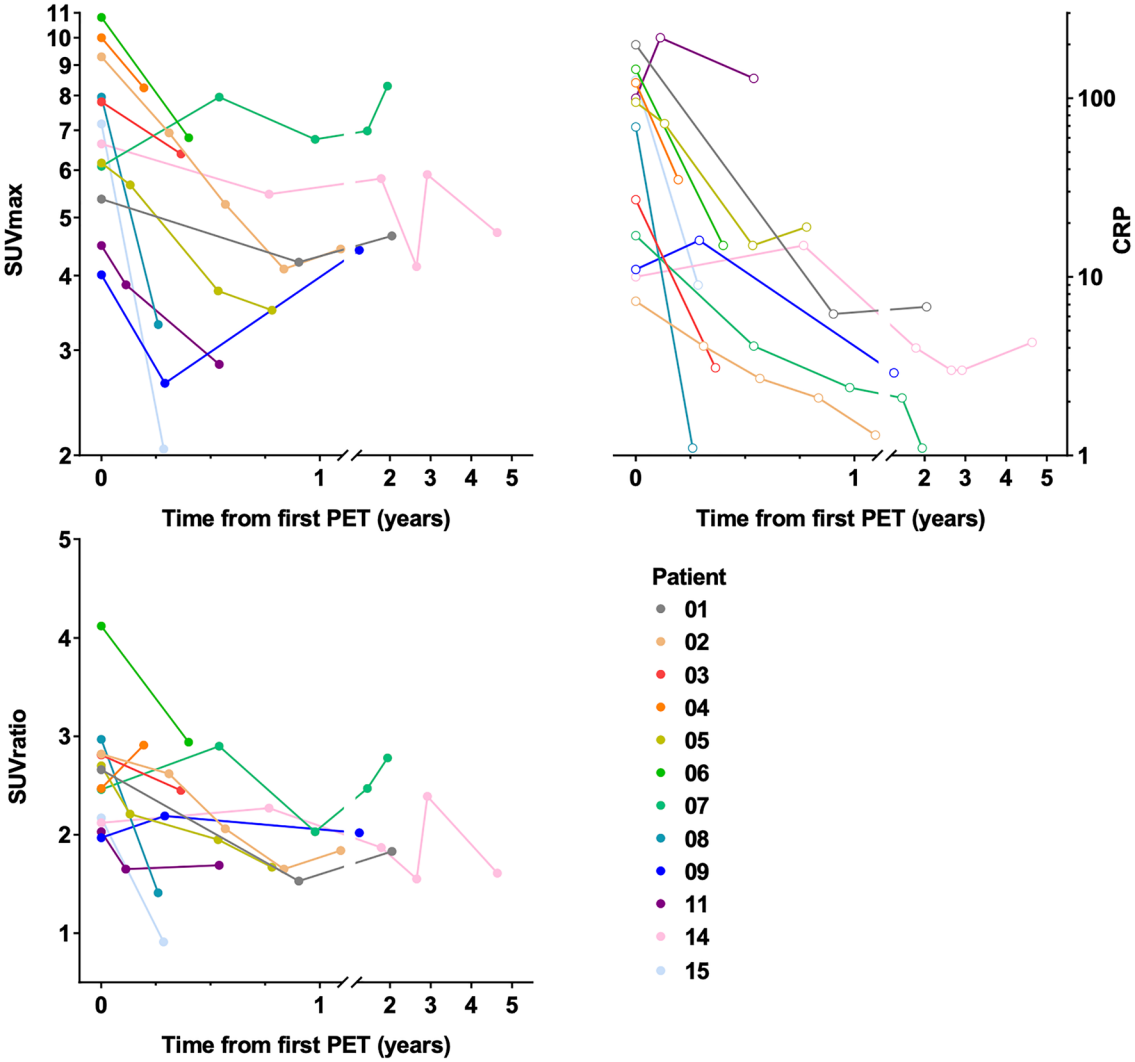
Courses over time of metabolic activity in PET/CT (SUVmax and SUVratio in the two graphs on the left) and CRP (graph on the right) in patients with proven infective native aortic aneurysms. *Note*: PET/CT provides additional or altering information (as defined in the methods section) on the course of disease in at least 14 comparisons (56%) in 11 patients (73%), as compared to CRP alone (i.e. patients: 02, 03, 04, 05, 06, 07, 08, 09, 11, 14, 15). Abbreviations: PET: positron emission tomography; SUV: standardized uptake value; CRP: C-reactive protein.

The study design for all consecutively and prospectively enrolled patients included consecutive PET/CT scans, at baseline, during follow-up on antimicrobial therapy, and at the end of antimicrobial treatment. If feasible, a control PET/CT three months after the end of antimicrobial therapy was performed, to document continuous cure or possible signs for recurrence of infection. For all retrospectively enrolled patients, PET/CT was performed depending on the clinical situation of the patient; the reasons for patient’s referral are given in the results section. The exact time points of follow-up examinations with regard to the baseline are demonstrated in Fig. 1. For all patients, baseline was defined as first PET/CT scan at diagnosis of INAA.

We performed clinical follow-up of all patients by reviewing electronic patient charts. Patient data were recorded at the time of imaging, and at the last recorded clinical visit (recorded until March 2020).

Recorded data at baseline and at follow-up included patient demographics, clinical information, laboratory data (e.g. level of CRP and leucocyte count), results of microbiology, results from other diagnostic procedures, and information about treatment.

### PET/CT data acquisition and image analysis

Five different types of PET/CT scanners were used within the study period between 2005 and 2018, i.e. a Discovery ST16, a Discovery VCT, two Discovery MI a Discovery 690 and a Discovery 710 (all GE Healthcare, Waukesha, WI). Body weight, height, and blood glucose level were measured prior to imaging (blood glucose levels < 12 mmol/l were accepted^13^). All PET/CT examinations followed basic study protocols: patients fasted for at least four hours, body-weight adjusted intravenous injection of FDG^14^, standardized uptake time of 60 min in supine position, non-enhanced CT scans for attenuation correction, data acquisition with arms overhead whenever possible.

All PET/CT examinations were independently analysed by two double board certified radiologists and nuclear medicine physicians, blinded to all clinical patient data. Readers determined the FDG uptake pattern in the aneurysm or graft to be focal or diffuse^15^. A consensus reading was performed if results differed. Furthermore, both readers quantified the FDG-uptake in the aneurysm or in the vascular graft by measuring the maximum standardized uptake value (SUVmax) in the aneurysm or graft as well as in the liver and mediastinal bloodpool (the latter as background for reference). In case SUVmax measurements were not identical among both readers concerning the measurements in the aneurysm or graft, a consensus reading was performed; in case of discordant SUVmax values for the background measurements, the mean was calculated. To compensate for differences in the sensitivity of the different PET/CT scanner generations between the years 2005 and 2018, and resulting differences in quantitative PET parameters (i.e. SUVmax), we calculated relatively scanner-independent FDG uptake ratios (i.e. SUVratio was defined as uptake in the aneurysm of graft in relation to the mediastinal blood pool). To determine differences between the course of C-reactive protein and SUVratio we calculated trends between consecutive examinations, and defined an arbitrary cut-off of > 20% between differences in trends as relevant.

Furthermore, for all baseline and follow-up PET/CT examinations, readers determined whether a INAA or a VGI was present or not, using a visual 4-point grading score. Score 1 (no signs for infected aortic aneurysm/VGI) and score 2 (most likely a non-infected aneurysm/VGI) were considered negative for infected aortic aneurysm, while score 3 (suspicion of infected aortic aneurysm/VGI) and score 4 (clear signs of infected aortic aneurysm/VGI) were considered positive for infection.

### Statistical analyses

Variables were expressed as median and interquartile range (25th, 75th percentiles) or percentages. Kaplan–Meier estimates were used to describe survival at 1 and 5 years. We compared sizes and visual grading scores of INAA from initial and last CT scans using nonparametric pairwise Wilcoxon signed-rank test. Mixed-effects multilevel linear regression were applied to analyze the individual changes of SUVmax, SUVratio and CRP. Statistical analysis were performed using commercially available software (Stata/SE, Version 15.1, StataCorp, College Station, Texas).

## Results

### Patient population

Seven out of 21 (33%) patients with confirmed INAA were prospectively enrolled, while 14 (66%) patients were retrospectively included. Five out of 14 retrospective patients were excluded due to lack of written consent and one out of the seven prospective patients was excluded due to lack of clinical follow-up after the first PET/CT.

Thus, the final patient population consisted of 15 patients. All patients were eventually treated with open and/or endovascular repair (six prior to the first PET/CT, seven prior to the second, and two prior to the third PET/CT) (Table 1).

**Table 1:** Patient demographics, data on interventional procedures, and patient outcome data of the final study population in patients with proven infective native aortic aneurysms.

Pat	Age	Vascular surgery	Surgical technique	Location of aneurysm	Reoperation	Outcome			
						Days after initial operation	Days after end of antimicrobial treatment	Status	Signs for recurrence
01	62	Y-Graft	Open	Infrarenal aorta	Second look	1938	1832	Cured	No
02	58	Y-EAP	Endovascular	Infrarenal aorta	n.a	1198	826	Cured	No
03	64	T-EVAR debranching	Endovascular	Aortic arch	n.a	1451	1346	Cured	No
04	70	T-EVAR debranching	Endovascular	Aortic arch	n.a	287	n.a	Ongoing*	n.a
05	82	T-EVAR	Endovascular	Descending thoracic aorta	n.a	758	643	Cured	no
06	48	Acute bridging EVAR bifurcation prothesis	Hybrid	Infrarenal aorta	Resection of EVAR Extraanatomic Y-graft Open abdomen treatment	579	334	Cured	No
07	50	TEVAR Renovisceral debranching	Hybrid	Supra-/juxta-renal aorta	Resection of parts of the duodenum due to fistula VAC-on-vessel	2585	n.a	Ongoing	Stable disease
08	76	T-EVAR	Endovascular	Descending thoracic aorta	n.a	1289	1183	Cured	No
09	56	Acute bridging EVAR	Hybrid	Iliac artery	Resection of EVAR Extraanatomic Y-graft Open abdomen treatment	2926	2508	Cured	No
10	71	Debranching A. mesenterica superior and T. coeliacus EVAR	Hybrid	Descending thoracic aorta	Prognostic laparoscopy due to peri-aortic abscess	1059	293	Cured	No
11	52	TA-EVAR	Hybrid	Descending thoracic aorta/suprarenal	Viscerales debanching	227	n.a	Cured	No^†^
12	40	Y-EAP	Endovascular	Infrarenal aorta	n.a	2798	n.a	Cured	No^‡^
13	61	Renoviscerales debranching Graft implantation	Open	Juxtarenal aorta	Open abdomen treatment	3163	3109	Cured	No
14	61	Graft implantation	Open	Infrarenal aorta	Open abdomen treatment	1919	n.a	Ongoing	Stable disease
15	85	EVAR	Endovascular	Infrarenal aorta	n.a	1931	804	Cured	No

Pat: Patient number; na, not applicable.

*Patient died due to gastrointestinal bleeding, no relation to thoracic INAA.

^†^Patient developed 1.5 years later an infected pancreatic cyst.

^‡^The active intravenous drug user died 8 years later due to aortic valve endocarditis.

At the time of the initial PET/CT examination, patients had a median age of 61 years (IQR 54–85), one patient (7%) was female, six (40%) were smokers or had a history of smoking, two (13%) patients were diabetic, and six (40%) had renal insufficiency. The diagnoses of INAA were confirmed by blood culture (n = 11; 73%), culture or PCR from tissue obtained during surgical revision (n = 2; 13%) and serology (n = 2, 13%) (Table 2).

**Table 2:** Data on microbiology and antimicrobial of the final study population in patients with proven infective native aortic aneurysms.

Pat	Microbiology*	Antimicrobial treatment				
		Intravenous	Oral	Strategy	Duration (days)	Complications
01	*Escherichia coli*	Piperacillin/tacobactam	Ciprofloxacin	Prolonged	136	None
02	*Streptococcus agalactiae*	Cetriaxone	Amoxicillin/clavulanic acid	Prolonged	382	None
03	*Candida albicans*	Caspofungin	Fluconazol	Prolonged	192	None
04	*Streptococcus agalactiae*	Penicillin	Clindamycin	Prolonged	289	Port infection
05	*Streptococcus agalactiae*	Ceftriaxone	Clindamycin	Prolonged	220	None
06	*Staphylococcus aureus*	Flucloxacillin	Ciprofloxacin Rifampicin	Prolonged	261	None
07	*Streptococcus gallolyticus* *Candida albicans* *Pediococcus acidilactici*	Daptomycin Ertapenem	Fluconazole Amoxicillin	Lifelong	Infinite	None
08	*Streptococcus pneumoniae*	Ceftriaxone Gentamicin	Moxifloxacin	Prolonged	122	Vestibular toxicity due to gentamicin
09	*Bacteroides thetaiotaomicron/ Clostridium perfringens*	Piperacillin/tacobactam	Amoxicillin/clavulanic acid Ciprofloxacin	Prolonged	458	None
10	*Coxiella burnetii*	na	Doxycycline Plaquenil	Prolonged	783	None
11	*Streptococcus pneumoniae*	Ceftriaxone Gentamicin	Clindamycin	Prolonged	118	None
12	*Staphylococcus aureus*	Flucloxacillin	Ciprofloxacin Rifampicin	Prolonged	176	None
13	*Salmonella typhi*	Ceftriaxon	Ciprofloxacin	Prolonged	99	None
14	*Coxiella burnetii*	na	(Doxycycline) (Plaquenil) Minocycline	Lifelong	Infinite	Allergy to doxycyclin
15	*Porphyromonas gingivalis*	Penicillin	Clindamycin	Prolonged	1139	None

Pat: Patient number; na, not applicable.

*Microbiology resulted from blood culture (n = 11), culture or PCR from tissue obtained during surgical revision (n = 2) and serology (n = 2).

#### PET/CT examinations

**Figure 2:**
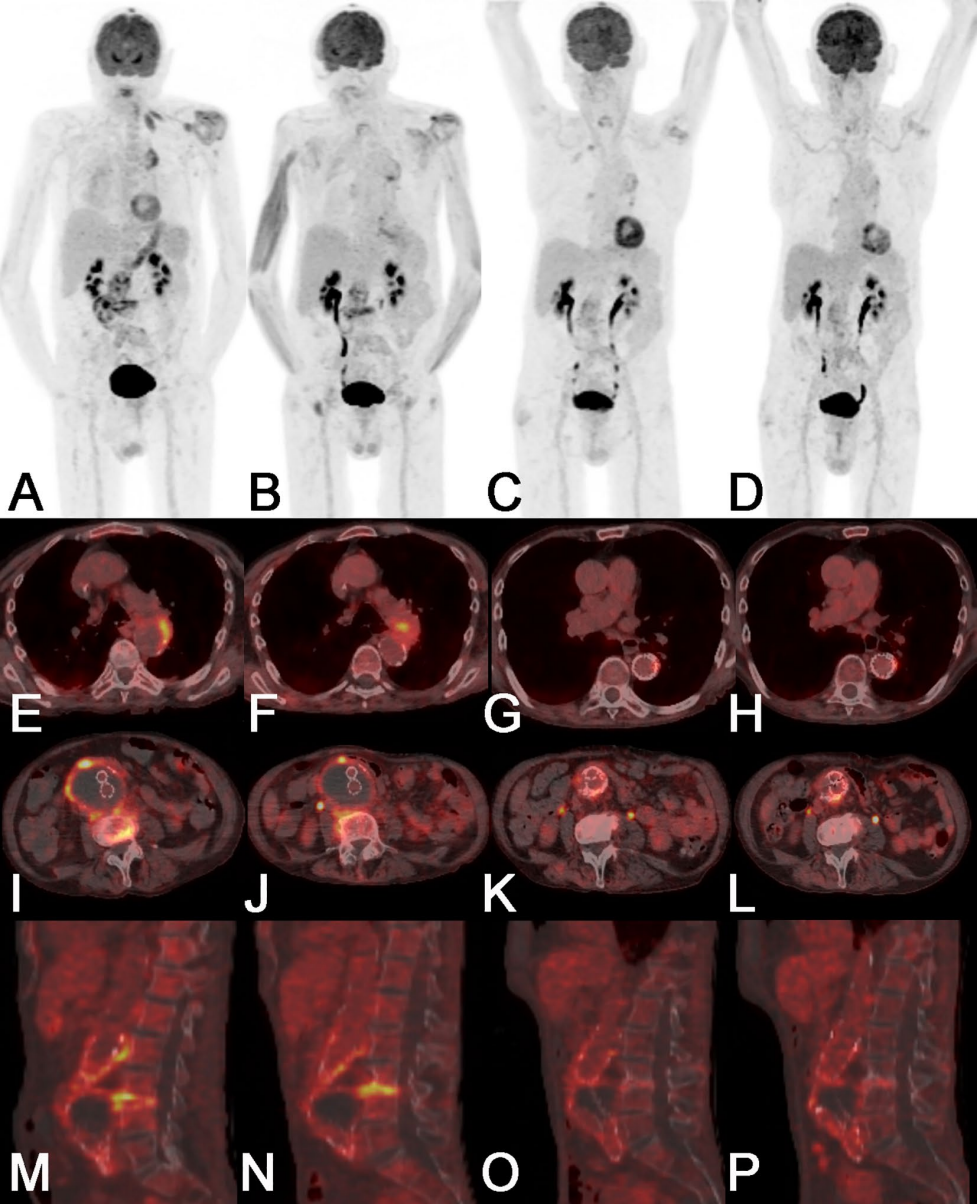
PET/CT of an 82-year old male patient (patient 05 in Tables 1, 2 and 3) with a vascular graft infection and spondylodiscitis due to *Streptococcus agalactiae* showed a new focal FDG uptake in the wall of the thoracic aorta in September 2017. Both readers rated the thoracic finding as a infective native aneurysm despite the fact that the vessel diameter was not pathologically widened. The first PET/CT follow-up in revealed a progression of the aneurysm in size with stable increased FDG uptake; at the same time the FDG uptake of the spondylodiscitis increased while it partially decreased in the vascular graft infection; C-reactive protein and white blood cell count decreased. After subsequent thoracic endovascular repair with an Endurant II Stent Graft Systems (MEDTRONIC), two further PET/CT follow-up, before and after termination of antimicrobial therapy (223 days of therapy) showed faint residual FDG uptake in all sites of infection. At the last clinical follow-up in January 2019 the patient was in good clinical condition with no sign of infection. *Note*: Panels A-D show maximum intensity reconstructions of PET; Panels E-P show fused PET/CT images. Abbreviations: PET: positron emission tomography; CT: computed tomography; FDG: 18F-fluorodeoxyglucose.

**Figure 3:**
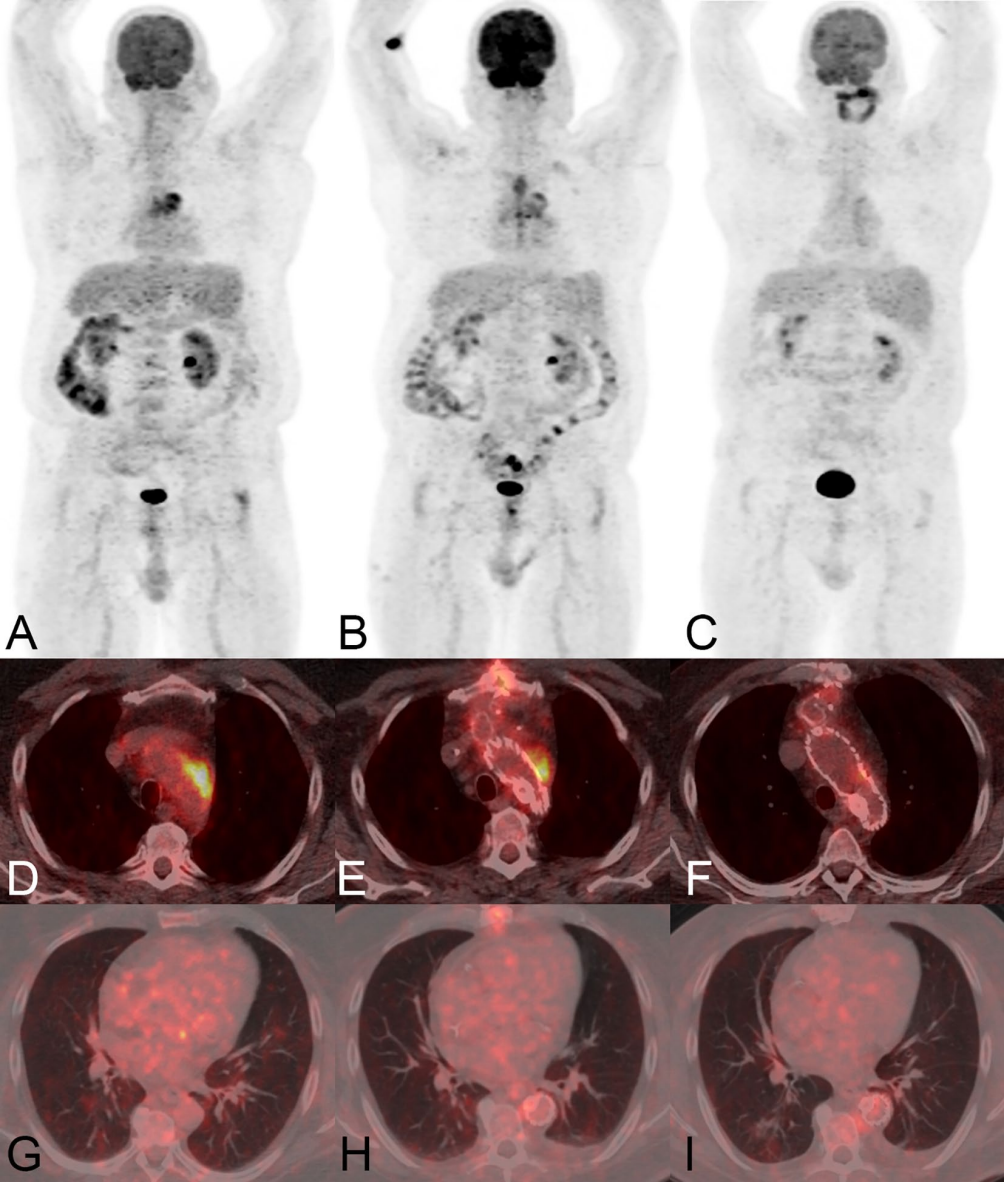
A 70-year old male patient (patient 04 in Tables 1, 2 and 3) presented with chest pain and signs for infection (C-reactive protein 122 mg/L). The initial PET/CT examination showed strongly increased FDG-uptake in the wall of an aortic arch aneurysm and no other infectious foci. Both PET/CT readers suspected a infective native aneurysm; which was clinically confirmed (*Streptococcus agalactiae* in blood cultures). At first PET/CT follow-up (ongoing antimicrobial therapy, after thoracic endovascular repair with a Conformable GORE TAG Thoracic Endoprosthesis and debranching) a strong residual focal FDG-uptake was detected adjacent to the graft, in line with a secondary vascular graft infection. The second PET/CT follow-up (ongoing antimicrobial therapy), showed only very faintly increased FDG-uptake adjacent to the graft. However, a new pneumonia in the right lower lobe was incidentally detected. The patient died 60 days after the last PET/CT due to a gastrointestinal bleeding not related to the thoracic infective native aortic aneurysm. *Note*: Panels A-C show maximum intensity reconstructions of PET; Panels D-I show fused PET/CT images. Abbreviations: PET: positron emission tomography; CT: computed tomography; FDG: 18F-fluorodeoxyglucose.

We performed a total of 53 PET/CT scans in 15 patients (Figs. 1, 2, 3, Table 3) after intravenous injection of a median of 353 Megabecquerel of FDG (IQR 334—400). Initial imaging was performed for nine (60%) patients before and for six (40%) patients after vascular intervention, 11 (73%) received antimicrobial therapy at the time of initial imaging. The median visual 4-point grading score was 4 (11 × score 4, 3 × score 3, 1 × score 2). Eventually, all patients underwent vascular intervention with graft placement, and were treated with antibiotics (Tables 1 and 2). All aneurysms (or grafts) had increased focal FDG uptake on the initial PET/CT examination (SUVmax 6.8 (IQR 5.7–21.8); SUVmax aneurysm/graft to blood pool background ratio 2.7 (IQR 2.2–7.3)). FDG uptake in all aneurysms/grafts (100%) was higher than liver background (SUVmax 3.4 (IQR 2.7–6.1) (Figs. 1, 2, 3, Table 3).

**Table 3:** Patient demographics, follow-up PET/CT and CT findings of the final study population in patients with proven infective native aortic aneurysms.

Pat		PET/CT						Initial morphologic signs in CT	Morphologic signs at last CT
		1	2	3	4	5	6	Size INAA in cm visual imaging score	Size INAA in cm visual imaging score
01	SUVmax CRP (mg/L)	5.4 199	4.4 n.a	4.3 n.a	4.2 6	4.7 7		*Stranding, fluid, gas	^†^Stranding
	Days to start of AB Days to end of AB	− 3 133	− 112 24	− 230 − 94	− 328 − 192	− 740 − 604		7.1 4	3.4 3
02	SUVmax CRP (mg/L)	9.3 7	6.9 4	5.3 3	4.1 2	4.4 1		^†^Stranding, fluid, CE	*Lymph
	Days to start of AB Days to end of AB	− 79 303	− 192 190	− 284 98	− 380 2	− 475 − 93		6.7 4	4.3 3
03	SUVmax CRP (mg/L)	7.8 27	6.4 3					^†^Stranding, CE, lymph	^†^None
	Days to start of AB Days to end of AB	− 45 147	− 175 17					2.7 4	Not measurable 3
04	SUVmax CRP (mg/L)	10 122	8.2 35	3.9 n.a				^†^Stranding, CE	*None
	Days to start of AB Days to end of AB	6 n.a	− 64 n.a	− 230 n.a				4.6 4	Not measurable 3
05	SUVmax CRP (mg/L)	6.2 95	5.7 72	3.8 15	3.5 19			^†^Stranding, CE, lymph	^†^Stranding, lymph
	Days to start of AB Days to end of AB	− 21 199	− 68 152	− 214 6	− 303 − 83			4.4 4	4.4 3
06	SUVmax CRP (mg/L)	11 145	6.8 15					^†^Stranding, CE, lymph	*Stranding, fluid, CE, gas, lymph
	Days to start of AB Days to end of AB	− 3 258	− 148 113					6.6 4	4.9 3
07	SUVmax CRP (mg/L)	6.1 17	7.9 4	6.8 2	7.0 2	8.3 1	7.9 n.a	*Stranding, fluid, gas	*Stranding, fluid, gas
	Days to start of AB Days to end of AB	− 818 n.a	− 1011 n.a	− 1170 n.a	− 1337 n.a	− 1515 n.a	− 1702 n.a	3.3 3	3.6 4
08	SUVmax CRP (mg/L)	7.9 69	3.3 1					^†^Stranding, fluid, CE	*Stranding
	Days to start of AB Days to end of AB	− 6 116	− 101 21					5.5 4	3.1 3
09	SUVmax CRP (mg/L)	4.0 11	2.6 16	3.9 n.a	2.0 n.a	4.4 3		*Stranding, lymph	*Stranding
	Days to start of AB Days to end of AB	− 71 387	− 176 282	− 325 133	− 421 37	− 520 − 62		2.7 3	2.7 2
10	SUVmax CRP (mg/L)	22 31	5.7 n.a					^†^Stranding, fluid, CE	*Stranding
	Days to start of AB Days to end of AB	− 11 772	− 721 62					6.6 4	4.0 3
11	SUVmax CRP (mg/L)	4.5 100	3.9 218	2.8 129				^†^Stranding, fluid, CE	*Stranding
	Days to start of AB Days to end of AB	40 158	0 118	− 153 na				6.0 3	6.4 1
12	SUVmax CRP (mg/L)	6.8 146	2.4 n.a	2.6 n.a	2.0 n.a			*Stranding, fluid	^†^Lymph
	Days to start of AB Days to end of AB	− 4 n.a	− 98 n.a	− 464 n.a	− 2602 n.a			5.8 4	Not measurable 1
13	SUVmax CRP (mg/L)	1.1 23	2.3 n.a					^†^Stranding, fluid, CE, gas	*Stranding
	Days to start of AB Days to end of AB	− 20 79	− 163 − 64					4.1 2	3.3 2
14	SUVmax CRP (mg/L)	6.6 10	5.5 15	5.8 4	4.1 3	5.9 3	4.7 4	*Stranding, fluid	*Stranding, fluid
	Days to start of AB Days to end of AB	24 n.a	− 253 n.a	− 618 n.a	− 933 n.a	− 1027 n.a	− 1646 n.a	8.3 4	4.2 2
15	SUVmax CRP (mg/L)	7.2 126	2.1 9					*Stranding, fluid, lymph	Lymph
	Days to start of AB Days to end of AB	6 1145	− 96 1043					4.5 4	4.1 1

Pat: Patient number; AB: antimicrobial treatment; n.a.: not applicable; stranding: fat stranding; fluid: fluid collection; CE: contrast enhancement, gas: gas formation; lymph.: lymph adenopathy.

*Non-enhanced CT;^†^ contrast-enhanced CT.

The first follow-up PET/CT (112 (IQR 95–721) days after the first PET/CT) was performed in all patients, and 13 (87%) patients received antimicrobial therapy at the time (in one patient antimicrobial therapy was started after the second PET/CT in another therapy was already terminated). The median visual 4-point grading score decreased to 3 (3 × score 4, 8 × score 3, 2 × score 2, 2 × score 1). The first follow-up showed an overall decrease in SUVmax (5.5 (IQR 3.0–8.2) and SUVmax ratio (2.2 (IQR 1.6–2.9)); SUVmax decreased in 13 and increased in two patients, SUVmax ratio decreased in 10 and increased in five patients (Figs. 1, 2, 3, Table 3).

In 11 patients, PET/CT was performed prior to the termination of the antimicrobial therapy. Compared to background activity, PET/CT showed focally increased FDG uptake in nine patients (82%) and diffuse increased FDG uptake in two patients (18%) with SUVmax 3.9 (IQR 2.7–6.8) and SUV ratio 1.7 (IQR 1.2–2.9). The FDG uptake in the aneurysm/graft was higher than blood pool background in eight (73%) patients (Fig. 2) and higher than liver background in seven (64%) patients. The median CRP was 12.0 mg/L (IQR 3–218). Of note, the median time difference between the last PET/CT prior to the termination of antimicrobial therapy and the actual end of antimicrobial therapy was a median 30 days (IQR 18–1059), and six examinations (55%) were considered to be directly linked to the decision to end treatment.

A total of eight PET/CT were performed after termination of the antimicrobial therapy treatment in six patients (SUVmax in all eight PET/CT: 4.2 (IQR 3.3–4.7); SUVmax in the first six PET/CT 3.9 (IQR 3.0–4.4). Compared to background activity, six (75%) of these examinations showed increased and focal FDG uptake (SUVmax 4.2 (IQR 3.3–4.7), SUV ratio 1.7 (IQR 1.5–4.1)), in two examinations (25%) the uptake was considered diffuse; FDG uptake was higher than blood pool background (SUVmax 2.2 (IQR 2.0–2.9)) and liver background (SUVmax 3.2 (IQR 2.7–3.6)) in all examinations (100%). The median visual 4-point grading score after termination of the antimicrobial therapy was 3 (0 × score 4, 5 × score 3, 2 × score 2, 1 × score 1).

The comparison of the last PET/CT prior to the termination of antimicrobial therapy to the first PET/CT after termination of antimicrobial therapy (feasible in six patients) showed less focal FDG-uptake (from five to four) and decreasing FDG uptake in three patients for SUVmax and in two patients for SUVratio, while the uptake increased in three patients for SUVmax and in four patients for SUVratio (SUVmax from 3.8 to 3.9 (IQR 2.5–4.4 and 3.0–4.4) and SUV ratio from 1.6 to 1.7 (IQR 0.9–1.9 and 1.5–4.1)). Notably, the two patients with a significantly rising SUV ratio (from 0.7 to 2.0 and from 0.7 to 1.2; patient 9 and 13 in Table 3) had no signs of infection at the last clinical follow-up, which was more than five and more than eight years after the last PET/CT and without antimicrobial therapy. None of these patients had clinical signs of new VGI at the last clinical follow-up.

Sizes of INAA, measured on CT images, decreased significantly by 1.45 cm on average from the initial to the last examination (p = 0.016, Table 3). In parallel, visual grading scores for INAA decreased by 1.2 on average (p < 0.01, Table 3).

### Discrepancies between trends of SUVratio and CRP

With 53 PET/CT performed in 15 patients we were able to calculate 38 trends of SUVratio and 25 trends of CRP (immediate data of CRP missing for 13 PET/CT).

The decrease or increase of SUVratio was paralleled by decreasing or increasing CRP values in 16 of 25 (64%) trends, while it was opposed in nine (36%) examinations. Nineteen comparisons (76%) showed either opposed trends (as mentioned above, n = 9) and/or differences between trends of CRP and SUVratio > 20%. For these trends, PET/CT provided a conclusive explanation for the difference in five comparisons (26%) in three patients (i.e. additional foci of infection with different response to therapy: patient 05, 11, and 14 in Table 3 and Fig. 2). No apparent reason for high differences in trend could be determined for four comparisons (21%) in four patients (i.e. three opposed trends between first and second PET/CT in patient 04, 07, and 11 (Table 3) and a high 46% increase in CRP paralleled by a low 11% increase of SUVratio in patient 09 in Table 3). In five comparisons of five patients, CRP values showed very good or complete responses to therapy at the time of the first follow-up PET/CT, which were paralleled by less pronounced partial responses in SUVratio (patient 02, 03, 06, 08, and 15); further clinical follow-up could not determine which methods better described the actual clinical response to therapy (as all other clinical parameters defining cure of INAA were normal/unchanged). The final five comparisons of differences between trends of CRP and SUVratio which were > 20%, were rated to be errors as the values were very low and partly normal, i.e. between the fourth and fifth PET/CT of patient 02 (Table 3), CRP values decreased by 80% from 2.1 to 1.3, which was paralleled by a 20% increase in SUV ratio from 1.65 to 1.84.

Hence, PET/CT provided additional or altering information on the course of disease in at least 14 comparisons (56%) in 11 patients (73%), as compared to CRP alone.

### Patient outcome

Patients were clinically followed for a median of 1496 days (IQR 961–3187 days) after their initial PET/CT examination and for a median of 1005 days (IQR 683–3109 days) after their last PET/CT examination. The one-year and five-year freedom from all-cause mortality was 92% (95% confidence interval [CI] 57–99%), for both. Overall mortality was 3.5% (0.9–13.8) per year. Two events occurred in 57.8 years of follow-up time; i.e. two patients (13%) died because of reasons considered unlikely to be related to INAA; one died due to gastrointestinal bleeding (location of the INAA was in the aortic arch), the other due to aortic valve endocarditis (after more than six years after successful treatment of the INAA).

The two patients (13%) on continuous antibiotic therapy, showed higher metabolic activity in the INAA (SUVmax 7.9 and 4.7, SUVratio 2.4 and 1.6) than the mean metabolic activity of those patients that were scanned prior to the termination of antimicrobial treatment (as mentioned above: SUVmax 3.9 (IQR 2.7–6.8) and SUV ratio 1.7 (IQR 1.2–2.9)). The conditions of these two patients were determined to be stable at the last clinical visit 261, and 729 days after the last performed PET/CT.

One patient (7%) showed recurrent signs of infection at the last clinical visit, which were attributed to an infected pancreatic cyst. All other patients (n = 10, 67%) did not show any signs of infection at the last clinical visit and all were without antimicrobial therapy (Table 1).

Among patients alive without antimicrobial therapy, yearly changes of SUVmax, SUVratio and CRP were -3.5 (95% CI -5.5 to -1.5, p < 0.01), -1.0 (-1.7 to -0.3, p < 0.01) and -58 (-96 to -20, p < 0.01), respectively. Among patients still on antimicrobial therapy or who died, yearly changes of SUVmax, SUVratio and CRP were -0.39 (95% CI -0.81 to 0.03, p = 0.07), -0.20 (-0.33 to -0.08, p < 0.01) and -4.6 (-14.2 to 5.1, p = 0.35), respectively.

## Discussion

To the best of out knowledge, this is the first study investigating the role of PET/CT in therapy control of INAA. Our study results show: (i) PET/CT adds additional information in therapy control of INAA. (ii) Metabolic activity in the aneurysms remains slightly elevated after the end of antimicrobial therapy and therefore should not be mistaken for persistent infection.

All patients of the present study were treated with open and/or endovascular repair, and as this treatment in INAA frequently leads to secondary graft infections, we may compare the present study results to the findings of three previous publications^9–11^ on therapy control with PET/CT in VGI. In line with these studies^9–11^, we found that in addition to laboratory and clinical information, consecutive PET/CT examinations were a valuable source of information for treatment monitoring, often displaying additional and sometimes even opposed results to clinical and laboratory parameters.

Previous studies have documented a correlation between the course of CRP and SUVmax in therapy control of patients with VGI^10^. In the present study, only a minority of comparisons (i.e. 24%) of CRP and the metabolic activity in the aneurysm showed a similar course in therapy control of patients with INAA. The majority of cases displayed large discrepancies between trends of CRP and metabolic activity or even opposed courses. The latter, may partly be explained by additional foci of infection with different response to therapy, which was in line with results of a previous study, showing correlation between CRP and SUVmax only in a subpopulation of patients with VGI without additional infectious foci^9^.

However, we could identify another group of comparisons with no apparent reason for the large discrepancies between trends of CRP and metabolic activity, which may indicate, that CRP and metabolic activity in the aneurysm are two unrelated identities, which may have to be evaluated independently, when treating patients with INAA. Because of such discrepancies and the overall severity of INAA in general, we believe that in all patients with INAA, decisions for further therapy should always be based also on other parameters such as clinical aspects (general health status, fever, clinical signs of INAA and/or graft infection), and other laboratory parameters (CRP, ESR, leucocytes).

Furthermore, we documented in line with previous reports^16–18^, that the metabolic activity in INAA is initially high or very high, and comparable to the initial presentation of VGI in PET/CT^9–11,15,19–25^. Under antimicrobial therapy the metabolic activity generally decreases^9–11^. However, the metabolic activity remains above background level before and even after the end of medical treatment; despite the fact that we did not observe any cases of recurrent infection. High rates of residual metabolic activity were also observed in patient populations with VGI at the end of medical treatment. Husmann et al.^10^ observed a complete metabolic response to treatment in only 33% of patients with VGI, while we could not observe a complete response in any of our patients. The reason for this difference remains unclear, and may possibly be due to differences in the course of two similar but yet different diseases, or it may be due to bias as our study cohort was considerably smaller in number. Notably, residual metabolic activity (i.e. SUVmax values slightly above background level) in the aneurysms after the end of medical treatment should not be mistaken for persistence of infection in INAA, but may possibly represent a sterile ongoing inflammatory reaction, as none of our patients showed signs of recurrence during the follow-up of this series and an excellent patient outcome was observed.

Two patients died due to reasons not considered unrelated to INAA. The one-year and five-year freedom from all-cause mortality was 93%, and was much higher than in previously published studies, which found five-year survival rates between 53%^26^ and 55%^7^. Whether disease monitoring with PET/CT contributed to the excellent clinical outcome in the present study still remains unanswered. However, we could not find any other obvious systematic difference, which may account for the improved clinical outcome.

### Limitations of the study

The present study population is heterogeneous, with varying numbers of follow-up PET/CT examinations, as well as prospectively and retrospectively included patients, which precludes standardized PET/CT intervals. Furthermore, due to the lack of comparable data in the literature, we defined an arbitrary cut-off of > 20% between differences in trends of SUVratio and CRP as relevant.

Despite these limitations, the results of the present study appear consequential and should be confirmed in further studies.

## Conclusion

As compared to the course of C-reactive protein, PET/CT provides different and occasionally altering information in therapy control of INAA. Of note, metabolic activity in the aneurysms remains slightly elevated even after the end of antimicrobial therapy and should not be mistaken for persistent infection.
